# Small mammals in a biodiversity hotspot harbor viruses of emergence risk

**DOI:** 10.1093/nsr/nwae463

**Published:** 2024-12-17

**Authors:** Yun Feng, Guopeng Kuang, Yuanfei Pan, Jing Wang, Weihong Yang, Wei-chen Wu, Hong Pan, Juan Wang, Xi Han, Lifen Yang, Gen-yang Xin, Yong-tao Shan, Qin-yu Gou, Xue Liu, Deyin Guo, Guodong Liang, Edward C Holmes, Zihou Gao, Mang Shi

**Affiliations:** State Key Laboratory of Remote Sensing Science, Center for Global Change and Public Health, Faculty of Geographical Science, Beijing Normal University, Beijing 100875, China; Yunnan Provincial Key Laboratory for Zoonosis Control and Prevention, Yunnan Institute of Endemic Disease Control and Prevention, Dali 671000, China; Yunnan Provincial Key Laboratory for Zoonosis Control and Prevention, Yunnan Institute of Endemic Disease Control and Prevention, Dali 671000, China; Ministry of Education Key Laboratory of Biodiversity Science and Ecological Engineering, School of Life Sciences, Fudan University, Shanghai 200433, China; National Key Laboratory of Intelligent Tracking and Forecasting for Infectious Diseases, Shenzhen Campus of Sun Yat-sen University, Sun Yat-sen University, Shenzhen 518107, China; State Key Laboratory for Biocontrol, Shenzhen Campus of Sun Yat-sen University, Sun Yat-sen University, Shenzhen 518107, China; Shenzhen Key Laboratory for Systems Medicine in Inflammatory Diseases, School of Medicine, Shenzhen Campus of Sun Yat-sen University, Sun Yat-sen University, Shenzhen 518107, China; Yunnan Provincial Key Laboratory for Zoonosis Control and Prevention, Yunnan Institute of Endemic Disease Control and Prevention, Dali 671000, China; National Key Laboratory of Intelligent Tracking and Forecasting for Infectious Diseases, Shenzhen Campus of Sun Yat-sen University, Sun Yat-sen University, Shenzhen 518107, China; State Key Laboratory for Biocontrol, Shenzhen Campus of Sun Yat-sen University, Sun Yat-sen University, Shenzhen 518107, China; Shenzhen Key Laboratory for Systems Medicine in Inflammatory Diseases, School of Medicine, Shenzhen Campus of Sun Yat-sen University, Sun Yat-sen University, Shenzhen 518107, China; Yunnan Provincial Key Laboratory for Zoonosis Control and Prevention, Yunnan Institute of Endemic Disease Control and Prevention, Dali 671000, China; Yunnan Provincial Key Laboratory for Zoonosis Control and Prevention, Yunnan Institute of Endemic Disease Control and Prevention, Dali 671000, China; Yunnan Provincial Key Laboratory for Zoonosis Control and Prevention, Yunnan Institute of Endemic Disease Control and Prevention, Dali 671000, China; Yunnan Provincial Key Laboratory for Zoonosis Control and Prevention, Yunnan Institute of Endemic Disease Control and Prevention, Dali 671000, China; National Key Laboratory of Intelligent Tracking and Forecasting for Infectious Diseases, Shenzhen Campus of Sun Yat-sen University, Sun Yat-sen University, Shenzhen 518107, China; State Key Laboratory for Biocontrol, Shenzhen Campus of Sun Yat-sen University, Sun Yat-sen University, Shenzhen 518107, China; Shenzhen Key Laboratory for Systems Medicine in Inflammatory Diseases, School of Medicine, Shenzhen Campus of Sun Yat-sen University, Sun Yat-sen University, Shenzhen 518107, China; National Key Laboratory of Intelligent Tracking and Forecasting for Infectious Diseases, Shenzhen Campus of Sun Yat-sen University, Sun Yat-sen University, Shenzhen 518107, China; State Key Laboratory for Biocontrol, Shenzhen Campus of Sun Yat-sen University, Sun Yat-sen University, Shenzhen 518107, China; Shenzhen Key Laboratory for Systems Medicine in Inflammatory Diseases, School of Medicine, Shenzhen Campus of Sun Yat-sen University, Sun Yat-sen University, Shenzhen 518107, China; National Key Laboratory of Intelligent Tracking and Forecasting for Infectious Diseases, Shenzhen Campus of Sun Yat-sen University, Sun Yat-sen University, Shenzhen 518107, China; State Key Laboratory for Biocontrol, Shenzhen Campus of Sun Yat-sen University, Sun Yat-sen University, Shenzhen 518107, China; Shenzhen Key Laboratory for Systems Medicine in Inflammatory Diseases, School of Medicine, Shenzhen Campus of Sun Yat-sen University, Sun Yat-sen University, Shenzhen 518107, China; National Key Laboratory of Intelligent Tracking and Forecasting for Infectious Diseases, Shenzhen Campus of Sun Yat-sen University, Sun Yat-sen University, Shenzhen 518107, China; State Key Laboratory for Biocontrol, Shenzhen Campus of Sun Yat-sen University, Sun Yat-sen University, Shenzhen 518107, China; Shenzhen Key Laboratory for Systems Medicine in Inflammatory Diseases, School of Medicine, Shenzhen Campus of Sun Yat-sen University, Sun Yat-sen University, Shenzhen 518107, China; State Key Laboratory of Respiratory Disease, National Clinical Research Center for Respiratory Disease, Guangzhou Institute of Respiratory Health, The First Affiliated Hospital of Guangzhou Medical University, Guangzhou 511436, China; Department of Arbovirus, National Key Laboratory of Intelligent Tracking and Forecasting for Infectious Diseases, National Institute for Viral Disease Control and Prevention, Chinese Center for Disease Control and Prevention, Beijing 102206, China; School of Medical Sciences, The University of Sydney, Sydney, New South Wales 2006, Australia; Laboratory of Data Discovery for Health Limited, Hong Kong 999077, China; Yunnan Provincial Key Laboratory for Zoonosis Control and Prevention, Yunnan Institute of Endemic Disease Control and Prevention, Dali 671000, China; National Key Laboratory of Intelligent Tracking and Forecasting for Infectious Diseases, Shenzhen Campus of Sun Yat-sen University, Sun Yat-sen University, Shenzhen 518107, China; State Key Laboratory for Biocontrol, Shenzhen Campus of Sun Yat-sen University, Sun Yat-sen University, Shenzhen 518107, China; Shenzhen Key Laboratory for Systems Medicine in Inflammatory Diseases, School of Medicine, Shenzhen Campus of Sun Yat-sen University, Sun Yat-sen University, Shenzhen 518107, China; Guangdong Provincial Center for Disease Control and Prevention, Guangzhou 510300, China

**Keywords:** wildlife virome, metatranscriptomics, zoonotic diseases, virus evolution, virus ecology

## Abstract

Metagenomic sequencing has transformed the understanding of viral diversity in wildlife and the potential threats these viruses pose to human health. Despite this progress, such sequencing studies often have lacked systematic and ecologically informed sampling, thereby likely missing many potential human pathogens and the drivers behind their ecology, evolution and emergence. We conducted an extensive search for viruses in the lungs, spleens and guts of 1688 mammals from 38 species across 428 sites in Yunnan Province, China—a hotspot for zoonoses emergence. We identified 162 mammalian viruses, including 102 new ones and 24 posing potential risks to humans due to their relationships with known human pathogens associated with serious diseases or their ability to cross major host species barriers. Our findings offer an in-depth view of virus organotropism, cross-host associations, host sharing patterns, and the ecological factors influencing viral evolution, all of which are critical for anticipating and mitigating future zoonotic outbreaks.

## INTRODUCTION

Most epidemics, including that of coronavirus disease 2019 (COVID-19), are zoonotic in origin, initiated by the transmission of a microorganism from animals to humans [1–4]. Factors such as unchecked wildlife exploitation, climate change and alterations in land use may amplify human exposure to novel pathogens, increasing the risk of zoonotic diseases [2,3,5–7]. Although predicting exactly which virus may emerge next is likely impossible [8,9], many viruses associated with human diseases had previously been identified in animal non-human hosts [10–13]. As a consequence, the surveillance of the viromes of animals that are closely linked to human populations is an important means to anticipate, mitigate and prevent future zoonotic outbreaks [2,9,14–17].

Advances in metagenomic sequencing, particularly total RNA sequencing (i.e. metatranscriptomics), have led to a better understanding of viral diversity and biology in animal populations [18–26]. Recent research focusing on mammals closely associated with humans, especially the rodents, the sympatric eulipotyphlans (e.g. shrews) and scandentians (i.e. treeshrews), has provided insights into potential zoonotic risks [23–26]. These animals inhabit varied terrestrial habitats with considerable ecological overlap with humans [27]. Some of these animals harbor major zoonotic pathogens such as the Lassa, lymphocytic choriomeningitis, and Hantaan viruses, and play important roles in the natural cycles of vector-borne viruses like Crimean-Congo hemorrhagic fever and tick-borne encephalitis viruses [28–30]. In addition, more structured metatranscriptomic surveys have informed virome ecology and evolution, revealing both known viruses in new hosts and novel viruses that pose a threat to human populations [26,31–34]. However, resource and

logistical constraints often limit the scope of mammalian surveillance geographically, temporally and numerically, resulting in a fragmented understanding of potentially emerging viruses and their ecological context.

Large-scale metagenomic studies often document virome compositions in host species at specific times and locations. This particularly holds true for studies of small mammals such as rodents and shrews. By contrast, recent virome analyses of bats and mosquitoes that incorporated a broader ecological context not only identified high-profile pathogens, but also provided important insights into the extent and pattern of cross-species virus transmission as well as the determinants of virus biogeography [24,35], in turn providing a clearer picture of the drivers of disease emergence.

Situated in a 394 000 km² area and home to more than one-half of China's plant and vertebrate species, Yunnan Province is a hotspot of global biodiversity [36]. Within this varied geographical landscape, we established extensive sampling sites across diverse environments to reveal the viromes of small mammals, including rodents, shrews and treeshrews, in doing so identifying viruses of potential zoonotic risk. Our data also address how both host and environmental factors impact virus richness, cross-species associations and genomic diversity.

## RESULTS

### Sampling of small mammals in Yunnan

**Figure 1.**
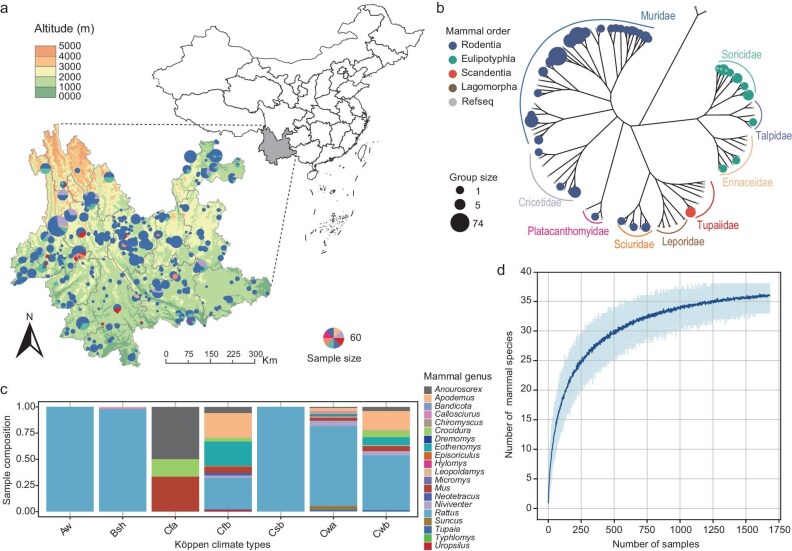
Distribution and diversity of small mammals in the study. (a) Map illustrating the sampling sites across Yunnan Province, China, color-coded by digital elevation in ArcMap 10.8.1 (the basemap shapefile was sourced from the publicly accessible USGS dataset). Pie charts at each site depict the mammalian family composition. (b) Phylogenetic tree based on the MT-CO1 gene of mammals analyzed in this study. Circle sizes reflect the number of sample groups and colors represent mammalian orders. (c) Composition of mammalian genera across different Köppen climate types, with segments color-coded to reflect the relative abundance of each genus. (d) Rarefaction curve demonstrating the relationship between the number of samples collected and the mammalian species identified. Review drawing number: GS 京(2025)0083号.

From 2021 to 2023, we conducted systematic animal sampling across Yunnan Province, China, capturing 1688 mammals from 428 locations across all 16 prefectures and 96 counties (Fig. 1a and b, Table S1). The elevations of these locations ranged from 144 to 3471 m, and our sampling covered seven of the nine Köppen climate types in the province, excluding only two high-altitude types in the northwest (Fig. 1a and c, Fig. S1). Each captured animal was analyzed using the mitochondrially encoded cytochrome c oxidase I (MT-CO1) gene, which confirmed the presence of 38 mammalian species representing 20 genera and eight families (Fig. 1b). A rarefaction analysis showed that the sampling effort was sufficient to capture the diversity of common small mammals examined here (Fig. 1d). Rodents, predominantly from the genus *Rattus*, were the most frequently captured, with 1540 individuals found at 419 sites. By contrast, shrews and treeshrews were relatively rare, with just 148 individuals from both groups captured across 49 sites. Notably, rats were widespread throughout the region, except in the warmest Cfa (humid subtropical) climate zone, where no samples were collected (Fig. 1c). Altitudinal analysis revealed species-specific distribution patterns, with Chevrier's field mice (*Apodemus chevrieri*) found at higher elevations and oriental house rats (*Rattus tanezumi*) at lower elevations (Fig. S1).

We strategically pooled 7–8 individuals from the same species and location into 207 sample groups for meta-transcriptomic sequencing, guided by mitochondrial sequence data (Table S2). For species represented by fewer than seven individuals from a specific region, samples were merged across broader areas, creating an additional 18 groups (Table S2; see Materials and Methods for details are provided in the supplementary data). Consequently, a total of 225 sample groups were organized. Metatranscriptomic libraries were prepared from organ tissues (gut, lung and spleen) for each group. A total of 646 libraries produced 21.99 billion clean non-rRNA reads, averaging 34.04 million reads per library (Table S2).

### Composition and diversity of mammalian virome

**Figure 2.**
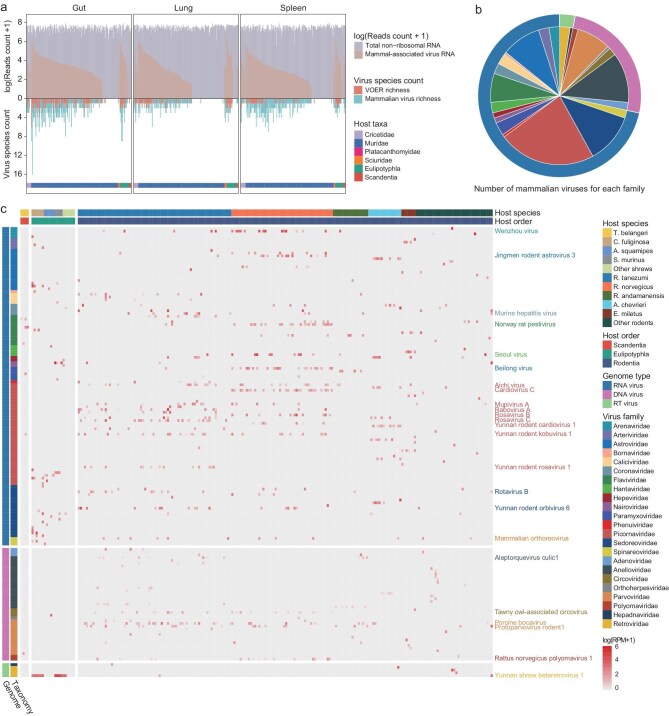
Overview of the diversity and prevalence of mammalian viruses identified in this study. (a) Total read depth, viral read abundance and species richness of viruses from each library, segmented by organ type: gut, lung and spleen. The libraries are organized first by host taxa and second by viral RNA abundance, arranged from high to low. (b) Pie chart showing the distribution of mammalian virus species identified within each virus family. (c) Heatmap of the distribution and relative abundance of mammalian viruses, quantifying viral abundance in each sample group by mapped reads per million non-rRNA reads (RPM). Host species and orders are labeled at the top, color-coded to match their respective categories. Viruses from 23 families are displayed, with each family distinctly colored; names are provided for only those viruses identified in more than 10 groups to emphasize the most prevalent one.

Our analysis revealed an extensive diversity of viruses across hosts. We focused on viruses associated with mammalian infections; specifically, those phylogenetically related to known (i) vertebrate-specific viruses and (ii) to vector-borne viruses that are associated with both mammals and arthropods (Fig. 2 and Fig. S2). Accordingly, we identified 5350 viral contigs, representing 162 mammalian-associated viral species across 23 families, including 116 RNA virus species from 16 families, 41 DNA virus species from seven families, and five reverse transcribing virus species from the *Retroviridae* and *Hepadnaviridae* (Fig. 2b). Notably, 102 viruses assigned to 19 families likely represent novel species according to the established International Committee on Taxonomy of Viruses species demarcation criteria (Table S3). Of the 646 libraries analyzed, 414 contained mammalian viruses, with viral RNA constituting from 1.08 × 10^−6^% to 15.43% of the total clean non-rRNA reads per library. Among these, the median number of mammalian viruses detected in each library was three, with an interquartile range of four, and a maximum of 16 distinct viruses (Fig. 2a). In total, 204 of the 225 sampled groups contained at least one mammalian virus. Rarefaction analysis, based on well-sampled mammalian species and virus richness, revealed that, with the exception of *Rattus norvegicus*, the viral richness in other species has not reached saturation (Fig. S3). This indicates that additional sampling is necessary to fully capture the viral diversity in small mammals.

The diversity and prevalence of viruses varied significantly depending on family. For instance, *Picornaviridae* was the most diverse, with 37 species identified, followed by the *Sedoreoviridae* and *Anelloviridae*, each with 19 species. By contrast, the *Bornaviridae* and *Phenuiviridae* were each represented by only one identified species (Fig. 2b). Commonly detected families included the *Picornaviridae, Sedoreoviridae, Flaviviridae* and *Parvoviridae*, found in at least 10 sample groups each, whereas families such as the *Bornaviridae, Hepadnaviridae* and *Orthoherpesviridae* appeared more sporadically (Fig. 2c).

### Identification of viruses of emergence risk

**Figure 3.**
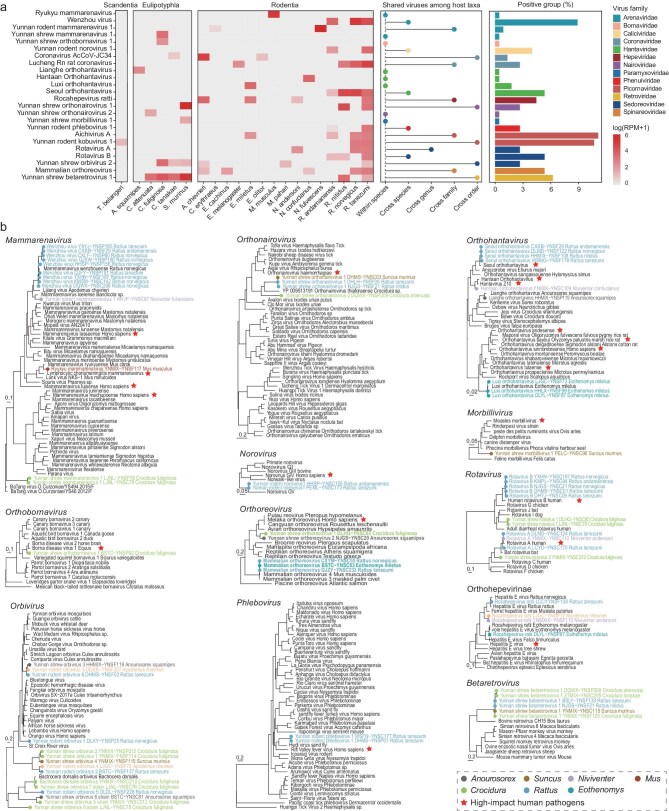
Phylogenetic relationships and epidemiological characteristics of viruses of emergence risk (VOER). (a) Mammalian host distribution, extent of virus sharing among hosts and prevalence of viruses. The heatmap illustrates the abundance of each virus within different host species, the middle bar chart depicts how viruses are shared among host taxa, and the right bar chart shows the prevalence of each virus. (b) Phylogenetic relationships and geographical distribution of VOER. Maximum likelihood phylogenetic trees for each established human-infecting virus genus were based on conserved viral proteins (RdRP for RNA viruses and reverse transcriptase for the *Retroviridae*). Trees were midpoint-rooted, with branch lengths representing the number of amino acid substitutions per site. Dots on the trees, colored according to host genera as indicated in the legend, represent viruses identified in this study. Previously identified human pathogens of each genus are marked with red pentagrams.

Among the 162 mammalian viruses identified here, 24 were designated as ‘viruses of emergence risk’ (VOER) based on their close phylogenetic relationships to known human pathogens (Table S4) and/or their increased risk of cross-species associations (Fig. 3a). Indeed, 20 of these VOER were closely related to known human pathogens (Fig. 3b, Table S4), highlighting their epidemic potential.

In the context of viral groups often associated with hemorrhagic fever or encephalitis, seven species of arenavirus and hantavirus were identified, including three that are newly discovered. Notably, Yunnan shrew orthobornavirus 1, was genetically very similar to classic *Borna disease virus 1* (87.6% amino acid identity of their large proteins) and *2* (88.5% identity), and was also related to *Variegated squirrel bornavirus 1* (74.8% identity), known to cause fatal human encephalitis [37] (Fig. 3b, Table S4).

In addition, we identified several new potential arthropod-borne viruses (i.e. arboviruses): one phlebovirus (Yunnan rodent phlebovirus 1) and two orthonairoviruses (Yunnan shrew orthonairovirus 1 and 2) that were closely related to *Rift Valley fever virus* and *Crimean-Congo hemorrhagic fever virus*, respectively, both of which can lead to lethal hemorrhagic fever. As such, these newly discovered viruses may also pose a threat to humans (Fig. 3b, Table S4). In addition, 15 species of orbivirus, again likely arboviruses, were discovered.

In the case of potential respiratory infections, we identified the new Yunnan shrew morbillivirus 1 that exhibited 66.3% sequence identity to *Measles morbillivirus*. Additionally, *rotavirus A* and *B*, new Yunnan rodent norovirus 1 and *Aichivirus A* were flagged as potential disease agents related to these enteric human viruses (Fig. 3b).

Several viruses were classified as VOER due to their capacity for transmission among phylogenetically distant mammalian species, demonstrating that they may represent host generalists (Fig. 3a). For instance, Lucheng Rn rat coronavirus and coronavirus ArcCoV-JC34 were identified in more than two mammalian families, whereas the new Yunnan shrew betaretrovirus 1 and Yunnan shrew orbivirus 2 were present in different mammalian orders. Interestingly, while not classified as VOER in this study, several viruses were found to be relatively closely related to viruses from different mammalian species. For example, Gibbon ape leukemia virus sampled from Chevrier's field mice exhibited strong sequence similarity with those isolated from gibbons (NC_001885, 94.90%), and Tawny owl-associated circovirus sampled from brown rats (*R. norvegicus*) was closely related to a sequence detected in the tawny owl (OL411978, 93.99%). These findings suggest potential host-switching events, or perhaps dietary associations in the case of the circovirus, although further data are needed to confirm this.

We also discovered viruses that occupy unique phylogenetic positions in the mammalian-associated virus lineages. For example, seven novel viruses carried by shrews fell as basal lineages to the genus *Orbivirus*, order *Reovriales*; Yunnan shrew mammarenavirus 1 is phylogenetically positioned between newly identified *BaTang virus* and the classic Old World and New World mammarenaviruses, whereas Yunnan shrew morbillivirus 1 fell basal to the entire genus *Morbillivirus* (Fig. 3b). Interestingly, these viruses were predominantly found in shrews from the genera *Anourosorex, Crocidura* and *Suncus*, suggesting that these taxa harbor diverse viromes.

### Organotropism of mammalian viruses

**Figure 4.**
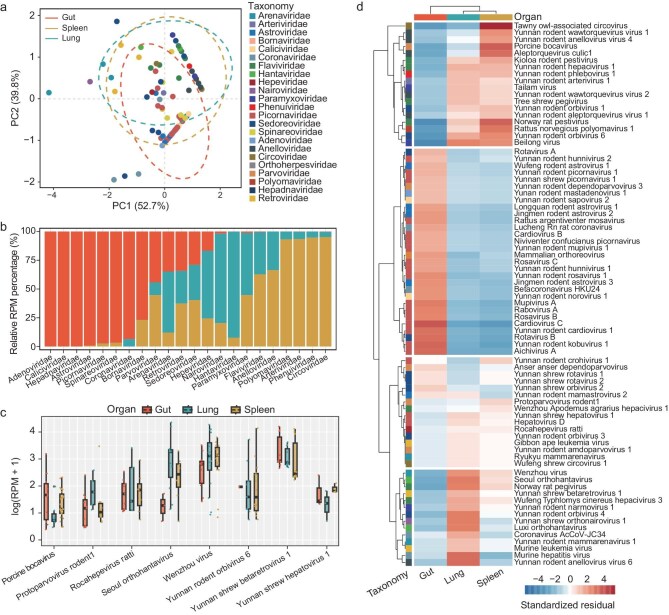
Organ distribution patterns of mammalian viruses. (a) Principal component analysis (PCA) based on the median viral abundance (RPM) of each viral species across different sample groups (colored by family), highlighting virome composition across the gut, spleen and lung. (b) Relative total viral abundance (RPM) for various viral families in gut (orange), lung (turquoise) and spleen (golden yellow). (c) Comparison of the abundance of viruses detected in at least three libraries for three organ types, with color keys shown in the legend. (d) Heatmap illustrating organotropism of viruses, determined by a chi-squared test analyzing distribution frequencies of viruses across guts, lungs and spleens.

Of the 225 groups sampled, 201 had a complete set of all three organs (guts, lungs and spleens), leading to 603 libraries that were used for organotropism analysis. Significant differences in virome composition and abundance were observed across these organs (adonis test, R² = 0.40, *P* = 0.001; Fig. 4a and Fig. S4). Additionally, an analysis of virus detection frequencies across different organs revealed significant variation in viral distribution (chi-squared test, χ² = 667.88, *P* < 0.05). Based on relative abundance, distinct organotropisms were noted for many viruses of viral families: *Hantaviridae* and *Nairoviridae* (primarily found in lungs), *Phenuiviridae, Arteriviridae* and *Circoviridae* (spleen), and *Picornaviridae, Caliciviridae* and *Coronaviridae* (gut) (Fig. 4b). Conversely, families such as the *Retroviridae, Arenaviridae, Hepeviridae, Parvoviridae* and *Sedoreoviridae* appeared in all three organs, suggesting systemic infections (Fig. 4b). Similar patterns of organ-specific and systemic infections were also noted at the level of viral species (Fig. 4c, d and Fig. S4). Interestingly, despite causing systemic infections, some viruses, such as minute virus of mice, Yunnan shrew betaretrovirus 1 and Yunnan shrew hepatovirus 1, despite causing systemic infections, had uneven abundance across different organs, indicating distinct organ preferences (Fig. 4d).

### Composition and multi-host associations of viruses

**Figure 5.**
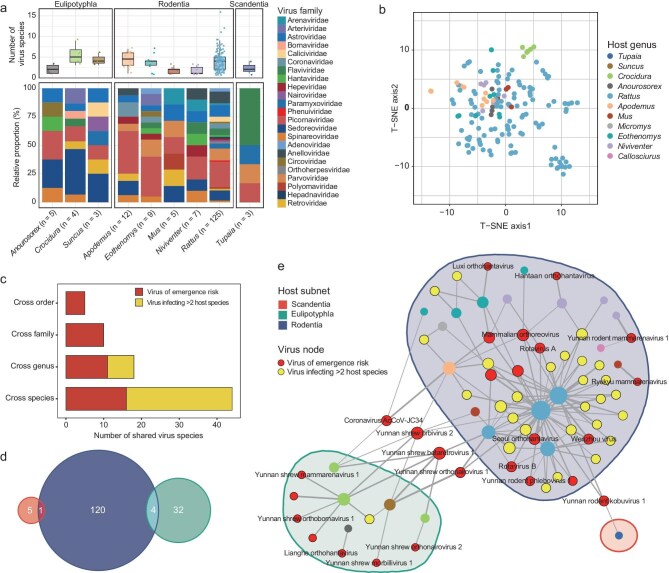
Viral composition and associations among mammalian hosts. (a) Observed number of viruses per sample group and their distribution across mammalian taxa. (b) t-SNE ordination illustrating the distinct virome compositions of mammals of different genera, with each point representing a group colored according to the host genus as indicated in the legend. (c) Number of viruses shared across species, genera, families and orders, highlighting extensive virological sharing among different mammalian hosts. (d) Venn diagram depicting specific viruses shared among the mammals of the orders Scandentia (red-orange), Eulipotyphla (teal) and Rodentia (dark blue). (e) Virus sharing network: nodes represent hosts or virus species, colored by host genera (aligned with 5b) and virus types. Line thickness between nodes indicates the relative abundance of viruses, with the network divided into three subnets based on host order. External nodes emphasize viruses with potential for cross-order associations.

To analyze virus composition and associations among different mammalian hosts, we focused on groups with complete data for three specific organs and excluded those with fewer than seven individuals or fewer than three sampled groups per genus. Consequently, from the orders Eulipotyphla (23 individuals, three groups), Scandentia (91 individuals, 12 groups) and Rodentia (1255 individuals, 159 groups), we identified six, 29 and 106 viruses, respectively (Fig. S4). Virus richness (i.e. number of viral species) varied markedly among host species (Fig. 5a): rodents primarily hosted members of the *Picornaviridae*; eulipotyphlans (i.e. shrews) mainly harbored viruses of *Sedoreoviridae*; and scandentians (i.e. treeshrews) commonly hosted members of *Flaviviridae*.

A t-distributed stochastic neighbor embedding (t-SNE) analysis visualized distinct virome compositions across host taxa (Fig. 5b and Fig. S5). PERMANOVA tests on viral genera with at least five sampled groups confirmed significant differences in virome compositions among mammals of different genera (n = 11, R² = 0.09, *P* < 0.001) and species from the same genus—specifically, three species of the genus *Rattus* (Fig. S5)—although the latter explained a small portion of variance (R² = 0.07, *P* < 0.001), indicating less pronounced differences at the intra-genus level.

We also identified several instances of virus sharing among different host taxa, indicative of multi-host species associations. A total of 44 viruses were detected in hosts of at least two host species, with 18 viruses found across different genera, 10 across different families and five across different orders (Fig. 5c). Scandentians and rodents shared one virus, while eulipotyphlans and rodents shared four (Fig. 5d). No viruses were shared among mammals of all three orders (Fig. 5e).

### Determinants of viral richness, composition and intra-specific genomic diversity

**Figure 6.**
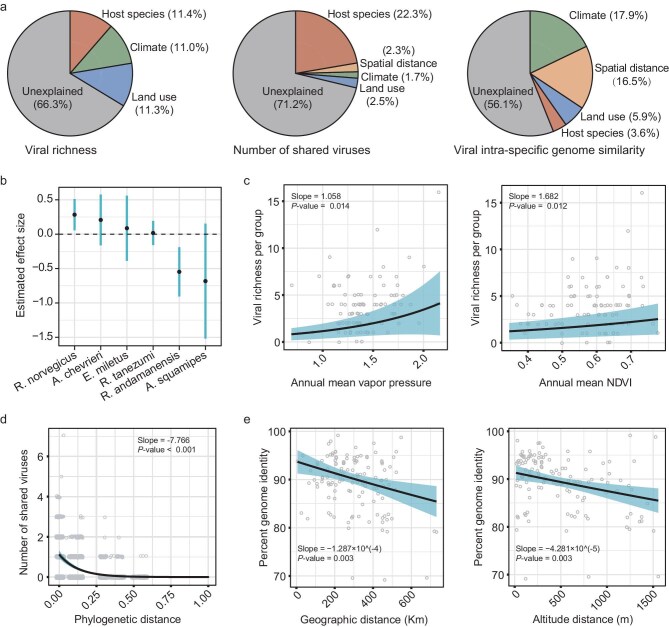
Key determinants of viral richness, composition and intra-specific genomic diversity. (a) Analysis of the relative contribution of host species, climate and land-use characteristics to viral richness in each sampled group, quantified by the explained deviance in the best model structures (ΔAIC < 2) using generalized linear models (GLMs). (b) Model-estimated effect size of mammalian species on viral richness per sampled group, presented with estimated values and 95% confidence intervals (CI). (c) Relationship of annual mean vapor pressure (a climate variable) and annual mean NDVI (a land-use variable) with viral richness in each sample group. (d) Relationship of the number of shared viruses and host phylogenetic distance (patristic distance based on the MT-CO1 phylogeny) among pairs of sample groups. (e) Impact of spatial distance (horizontal geographical distance and vertical altitudinal distances) on intra-specific viral genome similarity.

A total of 94 sample groups with suitable sample size and geographic range (see Materials and Methods for details are provided in the supplementary data) were selected for ecological comparisons to explore the environmental and host factors influencing viral richness (number of viral species), composition and genomic sequence diversity. Generalized linear models (GLMs) were utilized for these analyses. We selected the best model structures (ΔAIC < 2) by evaluating all combinations of variables based on the Akaike Information Criterion (AIC) (Fig. S6). This analysis identified that the key determinants varied among different metrics of viral diversity (Fig. 6a).

Variation in viral richness was influenced by host species affiliation (11.4%), climate (11.0%) and land-use variables (11.3%), with 66.3% of the total deviance remaining unexplained (Fig. 6a). Indeed, the distribution of viral richness across mammal species was uneven (Kruskal–Wallis H test, *P* < 0.05; Fig. 6b). Individual climatic variables, such as annual mean vapor pressure (slope = 1.058, standardized coefficient = 0.108, *P* = 0.014) and land-use variables like vegetation density (normalized difference vegetation index, NDVI) (slope = 1.682, standardized coefficient = 0.064, *P* = 0.012), positively influenced viral richness (Fig. 6c). Furthermore, separate analyses for the two most abundant rodent species—oriental house rats (n = 41) and brown rats (n = 20)—revealed that climatic conditions CPC1 (see Methods for details are provided in the supplementary data) and NDVI were the primary determinants, explaining 48.5% of the total variance in viral richness for oriental house rats. By contrast, for brown rats, viral richness was chiefly linked to human population density, accounting for 33.9% of the total variance (Fig. S6).

By contrast, similarity of viral composition, measured as the shared number of viral species among pairs of sampled groups (n = 94), were most significantly influenced by host phylogenetic distance (χ² = 686.52, *P* < 0.001), which explained 22.3% of the deviance in a GLM regression (Fig. 6a). After adjusting for other covariates such as climate differences, land-use variables and spatial distance, the number of shared viruses decreased as the phylogenetic distance between host groups increased (slope = –7.766, standardized coefficient = –2.130, *P* < 0.001; Fig. 6d). Conversely, the number of shared viruses was slightly negatively affected by differences in climatic variables (annual mean climatic water deficit), land-use characteristics (cropland presence) and mammal richness (Fig. S6).

Finally, intra-specific viral genetic diversity—defined as genome similarity within each viral species across all host species where the virus was detected—was mainly influenced by climate (17.8%) and spatial distance (16.6%). Notably, spatial distance emerged as a significant factor, unlike in the analysis of viral richness and composition. Indeed, viral genome similarity significantly decreased with increasing spatial distance, encompassing both horizontal geographical (slope = −1.287 × 10^−4^, standardized coefficient = –0.460, *P* = 0.003) and vertical altitudinal dimensions (slope = −4.281 × 10^−5^, standardized coefficient = −1.305, *P* = 0.003) (Fig. 6e and S6). By contrast, the association with host phylogenetic distance was relatively weak (slope = −4.957 × 10^−3^, standardized coefficient = −3.194 × 10^−6^, *P* = 0.96).

## DISCUSSION

Our study identified a number of viruses in rodents, shrews and tree shrews that may also have the capacity to emerge in humans, including both newly identified and previously known viruses associated with such syndromes as viral hemorrhagic fever, fever and myalgia, and encephalitis. This highlights the high risk of disease emergence at human–animal interfaces in Yunnan Province, China, where we found a greater variety of such zoonotic viruses than previously reported [26,31–34]. The well-established correlation between virus diversity and zoonotic risk with the ecological factors of reservoir richness, population size and density [21,38,39] supports these findings. Indeed, the viral diversity observed here can in large part be attributed to Yunnan Province's diverse geographical landscapes and its rich biodiversity. This region is globally recognized for its dense distribution of significant wildlife and is a critical terrestrial biodiversity area [36]. Additionally, our study benefits from the first broad and systematic sampling effort in this region, which covered a wide diversity of hosts and densely sampled areas. This comprehensive approach has deepened our understanding of which wildlife species and viruses pose significant risks to humans and clarified the context in which these viruses are likely to emerge.

Our discovery of numerous potentially zoonotic viruses is likely due to our comprehensive and targeted examination of three key organs—the lung, gut and spleen—each representing major systems (respiratory, digestive and circulatory) that serve as possible infection pathways through animal aerosols, excretions and shared arthropod vectors. This multi-organ approach has broadened our understanding of the range of pathogens posing threats to human health, overcoming the limitations of previous studies that targeted only specific tissues or swabs and likely overlooked significant portions of the virome [32,33]. Indeed, our data suggest that each organ likely harbors unique viral species. Interestingly, the spleen and lung shared more viruses with each other than with the gut. One likely explanation for this is that the spleen and lung share more cell surface proteins compared with the gut, primarily due to their roles in immune responses and the presence of similar immune cell populations. Our study also revealed that members of the families *Astroviridae, Caliciviridae, Coronaviridae* and *Picornaviridae* were predominantly found in the gut, whereas those of the *Flaviviridae* were primarily detected in the spleen, but rarely in feces. These findings are consistent with other virome studies of small mammals [26]. Interestingly, we detected a circovirus that was significantly enriched in the blood, differing from its typical manifestation in other mammals [40,41]. Additionally, our research identified viruses with multi-organ distributions, indicative of systemic infections, including several divergent members of the genus *Orbivirus*. The detection of these systemic infections highlights the critical role played by rodents and shrews as likely principal hosts for these viruses. Furthermore, systemic infections may also be associated with greater zoonotic potential due to diverse transmission routes.

Our study uncovered a large and intricate network of virus–host associations among small mammals in Yunnan Province, shedding light on potential zoonotic threats. On the one hand, we found that cross-species virus associations occur frequently, with 44 (27.16%) virus species carried by at least two host species. Conversely, our results also revealed strong host restrictions on cross-species associations, suggesting that the greater the genetic distance between hosts, the less likely it is that virus transmission will occur. This pattern was underscored by host genetic distance being the single dominant factor influencing viral composition, consistent with previous studies on other organisms [14,24,35,42,43]. Additionally, DNA viruses showed greater host specificity than RNA viruses [44]. Hence, although relatively frequent, most cross-species transmission events do not result in sustain transmission cycles in host hosts, such that spillover events to humans that result in zoonotic diseases are typically rare [17,21,45]. Nevertheless, some viruses demonstrate a notable capacity to infect genetically distant hosts: we identified 10 viruses present in at least three host species from different mammalian families, and four viruses in animals from different mammalian orders. These viruses, which we denote as VOER, merit particular attention due to their broad host range [14,23]. For instance, viruses capable of overcoming major host barriers, such as (Severe Acute Respiratory Syndrome Coronavirus 2) SARS-CoV-2—which uses the conserved ACE2 receptor to infect a wide range of mammalian species other than humans—highlight the great potential for zoonotic transmission [4,46,47]. Thus, the identification and close monitoring of such viruses are crucial for preventing zoonotic events.

Our extensive sampling across wide environmental gradients provided a robust dataset to investigate the ecological factors influencing viral diversity among mammalian wildlife. We found that viral richness, composition and intra-specific genetic diversity were driven by a combination of host species, climatic conditions and land-use variables. Specifically, viral richness was significantly influenced by host species diversity, annual mean vapor pressure and vegetation density, accounting for a considerable portion of the observed variance. While it is known that viral diversity may be affected by host, climate and land-use factors, such effects have seldom been quantitatively assessed. Our results provided a quantitative test of these effects, aligning with previous studies that highlight the role of environmental and ecological variables in shaping viral diversity. For instance, a previous study demonstrated that host taxonomy and land-use types (mountainous versus agricultural) were critical determinants of viral richness in bats, rodents and shrews [26]. However, in contrast to that study, we observed a greater frequency of viruses in rodents than shrews. Although our sampling was strongly biased towards rodents, diversity-hotspot hosts may vary spatially.

Our data also showed that inter-specific diversity (measured by the number of virus species shared among samples) and intra-specific diversity (measured by viral genomic sequence similarity among samples) are influenced by different factors. Inter-specific viral sharing is primarily driven by the phylogenetic distance between hosts, while climate and spatial distance have less or negligible influence. However, this analysis treats each virus as a whole without considering sequence variation within species. When focusing on genomic similarities within each viral species, differences in both climate and spatial distances emerge as the dominant factors, especially along vertical elevational gradients. This suggests that climatic and spatial factors do impact virus diversity, but that this influence is more pronounced on recent evolutionary scales. Despite this, overcoming spatial barriers is generally easier for viruses than overcoming host species barriers, with the latter clearly a major determinant of viral evolution [48,49].

### Limitations of the study

There were several limitations to our study. First, despite our findings, our understanding of the complete virome in small mammals remains incomplete due to the lack of data from other tissue types, such as the kidneys, which may harbor a distinct virome compared with the three tissues investigated in this study. Second, while the use of pooled samples for metatranscriptomic sequencing is practical for broad surveys, it may mask intra-species viral diversity, hinder the detection of low-abundance viruses, and limit the ability to examine host–virus interactions at the individual level. Third, there was also potential bias introduced by the uneven sampling across different climate zones and elevations. Although we attempted to capture a representative cross-section of mammals, certain areas, particularly the warmest and highest-altitude zones, were underrepresented. Another limitation is the focus on a specific geographic region, which may limit the generalizability of our findings to other areas with different ecological and climatic conditions. Finally, the emergence risk of newly identified viruses can only be preliminarily assessed through sequence and phylogenetic analysis here. To fully understand their virological characteristics and public health risks, further investigation through infection experiments is required. Despite these limitations, we were able to integrate ecological, climatic and host-related factors to elucidate the factors that shape viral diversity. This highlights the need for more extensive and geographically diverse studies to fully capture the global patterns of viral evolution and transmission.

## MATERIALS AND METHODS

The detailed descriptions of the materials and methods used in this study are provided in the supplementary data.

## Supplementary Material

nwae463_Supplemental_Files
