# Supplementary material for: Small mammals in a biodiversity hotspot harbor viruses of emergence risk
Source: Natl Sci Rev. 2024 Dec 17;12(6):nwae463. doi: 10.1093/nsr/nwae463 (PMC12151149; doi:10.1093/nsr/nwae463)
Supplement: nwae463_Supplemental_Files [file nwae463_supplemental_files.zip › nwae463_supplemental_file.docx]

Supplementary Information for

**Small mammals in a biodiversity hotspot harbor viruses of emergence risk**

Yun Feng^1,2, †^, Guopeng Kuang^2, †^, Yuanfei Pan^3^, Jing Wang^4,5,6^, Weihong Yang^2^, Wei-chen Wu^4,5,6^, Hong Pan^2^, Juan Wang,^2^ Xi Han^2^, Lifen Yang^2^, Gen-yang Xin^4,5,6^, Yong-tao Shan^4,5,6^, Qin-yu Gou^4,5,6^, Xue Liu^4,5,6^, Deyin Guo^7^, Guodong Liang^8^, Edward C. Holmes^9,10*^, Zihou Gao^2*^, and Mang Shi^4,5,6,11*^

**This file includes:**

Materials and Methods

Figures S1 to S6

**Materials and Methods**

**1. Study design and sample collection**

This study characterized the viromes of a broad spectrum of small mammal species to investigate the diversity of viruses they harbor and the ecological factors that shape virome composition. To ensure a representative sample across diverse ecological settings, 428 sampling sites (villages) across 96 counties in all 16 prefectures of Yunnan province, China, were systematically surveyed between 2021 and 2023. The number of sites varied from 1 to 22 per county, and from 4 to 108 per prefecture. These sites included both urban and rural areas, covered 7 of the 9 Köppen climate types (https://www.britannica.com/science/Koppen-climate-classification), and spanned altitudes from 144 to 3471 meters (Fig. 1a and S1, Table S1 and S2). In total, 1540 rodents, 125 shrews, and 23 treeshrews were captured (Table S3). Rarefaction analysis confirmed that the captured small mammals adequately represented the vast majority of the common species (Fig. 1d).

Rodents, shrews, and treeshrews were captured using snap-traps or cage-traps. Following capture, geographic coordinates and altitudes were recorded. Altitudes were then verified using latitude and longitude data with the basemap shapefile from the publicly accessible Earth Science Data Systems (ESDS). Animals were euthanized via intracardiac injection of sodium pentobarbitone and immediately transported on dry ice to the laboratory for dissection. Gut (large intestine with feces), spleen, and lung tissues were harvested, immediately preserved in dry ice or liquid nitrogen, and stored at -80°C prior to RNA extraction. All protocols for sample collection and processing were reviewed and approved by the Ethics Committee of the Yunnan Institute of Endemic Diseases Control and Prevention and Sun Yat-sen University (SYSU-IACUC-MED-2021-B0123).

Mammal species were initially identified by experienced field biologists based on morphological characteristics. This preliminary identification was confirmed by sequencing and analyzing mitochondrially encoded cytochrome c oxidase I (MT-CO1) gene for each specimen [1]. For each library, confirmation of mammalian species was achieved using *de novo* assembled contigs of MT-CO1 genes. The final clean MT-CO1 contigs were submitted to the BOLD online system [2] for species identification. A phylogenetic tree incorporating all full-length MT-CO1 sequences was estimated using PHYML 3.0 [3], employing the GTR+G nucleotide substitution model and the Subtree Pruning and Regrafting (SPR) branch-swapping algorithm.

**2. Sample groups**

Before RNA extraction, individual animals were first organized into sample groups. Typically, each group comprised 7–8 animals of the same species and location. In cases in which fewer than 7 animals were available from the same conditions, animals sampled from wider geographic areas (first from the same prefecture, then from the entire province) were combined into groups. Of these, 207 groups comprised 7–8 animals, among which 49 groups contained animals from exactly the same location, whereas 18 groups included fewer than 7 animals. In total, this approach resulted in 225 sample groups for the study (Table S2).

**3. RNA extraction, library construction and sequencing**

Tissue samples from each organ (gut with feces, lungs, and spleen) and each animal were individually homogenized in 600 µl of MEM solution (GIBCO). The homogenates were then pooled by organ type in equal volumes to create 200-µl pools for each sample group. As a result, each sample group had three distinct pools corresponding to the gut, lungs, and spleen. Total RNA was extracted and purified from each pool using the RNeasy Plus Universal Mini Kit (Qiagen, Germany). RNA libraries were then constructed using the Zymo-Seq RiboFree™ Total RNA Library Kit (No. R3003) according to the manufacturer’s instructions. These libraries were sequenced using paired-end (150-bp reads) on the Illumina NovaSeq 6000 sequencing platform.

**4. Identification and confirmation of mammalian viruses**

For the raw sequencing reads from each library, adapters were removed and initial quality control was conducted using the pipeline implemented in the bbduk.sh program (https://sourceforge.net/projects/bbmap/). The parameters for adapter removal included ktrim=r, k=23, mink=11, hdist=1, tpe, tbo. Quality control settings were maq=10, qtrim=r, trimq=10, ftl=5, minlen=90. Reads with extensive non-complex regions were excluded (parameters: entropy=0.5, entropywindow=50, entropyk=5). Duplicate reads were filtered out using cd-hit-dup under default settings [4]. rRNA reads were then removed by mapping the processed reads against the SILVA rRNA database (Release 138.1) using Bowtie2 (version 2.3.5.1) in the ‘--local’ mode [5]. The remaining high-quality, non-rRNA reads underwent *de novo* assembly with MEGAHIT (version 1.2.8) using default parameters [6]. The assembled contigs were then analyzed using DIAMOND BLASTx against the NCBI non-redundant protein database [7] with an e-value threshold of 1×10^-5^. Taxonomic classifications were assigned by correlating the top BLAST hit accession numbers to NCBI taxids, extracting those identified under the “kingdom” ‘Viruses’ for subsequent analyses.

Viral contigs shorter than 600 bp were excluded to ensure quality, and the remaining overlapping unassembled contigs were merged to form extended viral sequences using the SeqMan program implemented in the Lasergene software package (version 7.1, DNAstar) [8]. To verify genome integrity for viruses of families such as the *Retroviridae, Hepadnaviridae,* and *Bornaviridae* that may integrate into mammalian host genomes, open reading frames (ORFs) were identified using ORFfinder (https://www.ncbi.nlm.nih.gov/orffinder/) and sequences were classified via the online BLASTp program (https://blast.ncbi.nlm.nih.gov/Blast.cgi). The abundance of these viral contigs was estimated by mapping reads back to the assembled genome with Bowtie2 (version 2.5.2) using ‘--end-to-end’ and ‘--very-fast’ settings. Alignments were sorted and indexed with SAMtools (version 1.18) and visualized with Geneious Prime (version 2020.2.4) [9-10]. To further verify these findings and eliminate false positives, contigs were cross-referenced against the non-redundant nucleotide database using online BLASTn to exclude sequences related to the host genome, endogenous viral elements, and artificial vectors.

Each viral contig was classified at the species rank based on the species demarcation criteria established by the International Committee on Taxonomy of Viruses (ICTV) for the viral genus in question [11]. For genera lacking explicit species demarcation criteria, a 90% amino acid identity threshold for the RNA-directed RNA polymerase (RdRP) or replicase protein was applied. Viral contigs were further validated through comparisons of amino acid sequences from conserved genes, which included the RdRp for RNA viruses, pol for the *Retroviridae*, the major capsid protein for the *Orthoherpesviridae*, LTAg for the *Polyomaviridae*, ORF1 protein for the *Anelloviridae*, NS1 for the *Parvoviridae*, and DNA polymerase for other DNA viruses. Alignment of draft virus sequences and corresponding reference sequences from GenBank were performed using MAFFT (version 7.48) [12], with ambiguously aligned regions removed using TrimAl [13]. Phylogenetic trees were estimated using the maximum likelihood (ML) method implemented in PHYML 3.0 [3], employing the LG model of amino acid substitution and SPR branch-swapping. Only viral contigs that phylogenetically clustered with recognized mammalian-infecting viruses were considered mammalian viruses and retained for further analysis. Finally, 5,350 viral contigs were confirmed as mammalian viral sequences, from which 327 high-quality sequences were selected to represent the genomes of 162 mammalian viral species. These sequences have been uploaded to the CNGBdb (Table S3).

**5. Viral RNA quantification**

To quantify the amount of viral RNA present in our samples, we constructed reference sequences for all sequence variants of each viral species. Viral abundance was measured in each library by counting the number of viral reads per million of the non-rRNA reads (RPM). Reads were mapped to these reference genomes using Bowtie2 with ‘--end-to-end’ and ‘--very-fast’ settings, ensuring accurate alignment and quantification. To mitigate potential false positives from index-hopping, viral reads were only considered valid if they accounted for more than 0.1% of the highest read count within the same sequencing lane. Additionally, data characterized by low abundance (RPM <1) or insufficient genome coverage (< 300-bp) were excluded.

**6. Identification of viruses of emergence risk**

We used the term ‘virus of emergence risk’ (VOER) to designate viruses that likely pose a greater threat of emerging in human populations (i.e., zoonotic viruses), thereby distinguishing them from other, likely less impactful viruses. A virus is identified as a VOER if it meets any of the following three criteria: (i) phylogenetic clustering with established human pathogens or vector-borne virus groups at genus level, such as *Mammarenavirus, Norovirus, Orthornairovirus,* and *Morbillivirus*; (ii) that they were more than 80% identical in the deduced amino acid sequence encoded by conserved genes (RdRP or DNA pol) to those of known pathogenic viruses of humans; and (iii) evidence of frequent cross-species associations, demonstrated by the presence of the virus in question in at least three host species from different mammalian families.

**7. Virus distribution among organs within mammalian host species**

We also quantified the total abundance (RPM) and detection frequency of each virus across three organs (gut, lung, and spleen) to evaluate their possible tissue preferences. For robustness, only sample groups where all three organ types had been sampled and sequenced were included (Table S2). To enhance the robustness of our data, we excluded viruses detected in fewer than three libraries. The distribution data for each viral species across the organs were analyzed and visualized using the pheatmap package in R (version 4.1.1) [14]. We assessed the effect of organ type on virome composition, accounting for the potential influence of host species, using permutational multivariate analysis of variance (PERMANOVA) with the adonis2 function from the vegan package [15]. In this analysis, we represented virome composition with a Bray-Curtis distance matrix and used the median viral abundance (RPM) for each viral species. To investigate potential cross-species virus associations, we utilized network visualization in R (version 4.1.1) using the igraph package [16]. For the network visualization, the host-virus association matrix was collapsed into a virus-virus adjacency matrix based on shared host species.

**8. Collection of data for ecological comparisons**

To explore the impact of biodiversity and environmental factors on the diversity and composition of mammal-associated viromes, we gathered climate and land-use data for each sample site from public resources. Climate data were sourced from the TerraClimate data set [17], covering monthly climate variables—including six primary (Maximum temperature, minimum temperature, vapor pressure, precipitation accumulation, downward surface shortwave radiation, and wind-speed) and eight derivative metrics (Reference evapotranspiration, Runoff, Actual Evapotranspiration, Climate Water Deficit, Soil Moisture, Snow Water Equivalent, Palmer Drought Severity Index, and Vapor pressure deficit)—from 2021 to 2023. To reduce redundancy due to collinearity among climate variables, we conducted a principal component analysis. The first three principal components—CPC1, CPC2, and CPC3—accounted for 55.3%, 26.7%, and 6.3% of the total variance, respectively, cumulatively representing 88.3%. These components were subsequently utilized in all further statistical analyses to assess influences of climates on viral diversity and composition. Land-use data, obtained from the HYDE 3.2 database (5 arcmin spatial resolution) [18], were analyzed using the same PCA approach. The first two PCs, LPC1 and LPC2, explained 63.1% and 28.5% of the variance, respectively, totaling 91.6%. These components were then used to represent land-use patterns. Human population density data for 2022 were also sourced from the HYDE 3.2 database. Mammal richness data for each sampling site, aligned with the sampling year, were acquired from the International Union for Conservation of Nature mammal richness database [19]. Monthly NDVI data from NASA’s MODIS MOD13A3 product for the years 2021 to 2023 were downloaded and averaged annually to calculate annual average NDVI values.

**9. Statistic methods**

All statistical analyses were conducted using R version 4.1.1 [14].

**9.1 Group selection**

To minimize the confounding effects of uneven pooling sizes, we selected a subset of sample groups for our statistical analysis, focused on identifying the ecological drivers of viral diversity and cross-species associations. First, we included sample groups from mammalian species with at least three replications (i.e., > 3 sample groups). Second, we also restricted our analysis to groups containing lungs, spleen, and gut data as organ diversity can influence virus detection and composition. Third, to account for environmental variability within the same group, we ensured that the standard deviation for principal component scores of climate (CPC1, CPC2) and land-use (LPC1, LPC2) variables remained <1.2. Lastly, we also verified that the median, mean, and variance of distances among sampling sites within a group did not exceed 50 km. This selection process resulted in 94 groups that satisfied these conditions (Table S2), and all subsequent ecological analyses were performed based on this refined data set.

**9.2 Assessing viral richness and identifying key determinants**

We used generalized linear models (GLMs) with negative binomial regression, as implemented in the MASS package [20] in R, to analyze the impact of host species, climate, and land-use on viral richness. This analysis incorporated data on host species, climatic variables (CPC1, CPC2, CPC3), and land use metrics (LPC1, LPC2, NDVI, log-transformed population density, mammal richness), in addition to average altitude and site count. For groups derived from multiple sampling sites, numerical values such as CPC1, LCP1, NDVI, log-transformed human population density were averaged. Using the MuMIn package [21] in R, we systematically explored all possible variable combinations, selecting the most informative model based on the Akaike Information Criterion (AIC). This approach enabled a detailed assessment of each variable's specific contribution, providing a nuanced understanding of their individual and combined effects on viral richness across mammalian hosts.

**9.3 Comparisons of virus composition and sequence diversity and identifying key determinants**

We modeled the similarity in viral composition, defined as the number of shared viral species between sample pairs, using a generalized linear model (GLM). We quantified the relative effect of each factor, namely climate, land use, spatial distance, and host species, by the proportion of deviance explained and assessed the significance of each variable through single-term deletion from the model with a Chi-square test. Virome compositions across mammalian genera were visualized using t-SNE and differences were assessed through permutational multivariate analysis of variance (PERMANOVA), utilizing Jaccard distance calculated with the vegan package [15] in R.

Within species viral genomic diversity within species was evaluated by aligning all sequence variants for each species using ClustalW and pairwise sequence identities were computed with the msa package [22] in R. We also explored the impact of host phylogenetic distance, climatic and land use variations, and spatial distance on the virus sharing patterns and viral genomic diversity using generalized linear models (GLM). The specific influences of each variable were quantified employing a systematic approach similar to the previously described model selection and assessment methodology.

1. **Data availability**

The meta-transcriptomic sequencing reads generated in this study have been deposited in the China National GeneBank Database Sequencing Archive (CNSA) of China National GeneBank database (CNGBdb, https://db.cngb.org/cnsa; project accession: CNP0005966). The viral genome sequences generated in this study have been deposited in the CNGBdb under accession code N_AAJLEE010000000 to N_AAJLQS010000000 (Table S3).

**REFERENCE**

1. Ivanova, N. V., Dewaard, J. R., & Hebert, P. D. N.. (2010). An inexpensive, automation-friendly protocol for recovering high-quality dna. Mol.ecol.notes, 6(4), 998-1002.DOI:10.1111/j.1471-8286.2006.01428.x.
2. Ratnasingham, S., & Hebert, P. D. (2007). bold: The Barcode of Life Data System (http://www.barcodinglife.org). Molecular ecology notes, 7(3), 355–364. <https://doi.org/10.1111/j.1471-8286.2007.01678.x>
3. Guindon, S., Dufayard, J. F., Lefort, V., Anisimova, M., Hordijk, W., & Gascuel, O. (2010). New algorithms and methods to estimate maximum-likelihood phylogenies: assessing the performance of PhyML 3.0. Systematic biology, 59(3), 307–321. <https://doi.org/10.1093/sysbio/syq010>
4. Fu, L., Niu, B., Zhu, Z., Wu, S., & Li, W. (2012). CD-HIT: accelerated for clustering the next-generation sequencing data. Bioinformatics (Oxford, England), 28(23), 3150–3152. <https://doi.org/10.1093/bioinformatics/bts565>
5. Langmead, B., & Salzberg, S. L. (2012). Fast gapped-read alignment with Bowtie 2. Nature methods, 9(4), 357–359. <https://doi.org/10.1038/nmeth.1923>
6. Li, D., Liu, C. M., Luo, R., Sadakane, K., & Lam, T. W. (2015). MEGAHIT: an ultra-fast single-node solution for large and complex metagenomics assembly via succinct de Bruijn graph. Bioinformatics (Oxford, England), 31(10), 1674–1676. <https://doi.org/10.1093/bioinformatics/btv033>
7. Buchfink, B., Reuter, K., & Drost, H. G. (2021). Sensitive protein alignments at tree-of-life scale using DIAMOND. Nature methods, 18(4), 366–368. <https://doi.org/10.1038/s41592-021-01101-x>
8. Clewley J. P. (1995). Macintosh sequence analysis software. DNAStar's LaserGene. Molecular biotechnology, 3(3), 221–224. <https://doi.org/10.1007/BF02789332>
9. Li H. (2011). A statistical framework for SNP calling, mutation discovery, association mapping and population genetical parameter estimation from sequencing data. Bioinformatics (Oxford, England), 27(21), 2987–2993. <https://doi.org/10.1093/bioinformatics/btr509>
10. Kearse, M., Moir, R., Wilson, A., Stones-Havas, S., Cheung, M., Sturrock, S., Buxton, S., Cooper, A., Markowitz, S., Duran, C., Thierer, T., Ashton, B., Meintjes, P., & Drummond, A. (2012). Geneious Basic: an integrated and extendable desktop software platform for the organization and analysis of sequence data. Bioinformatics (Oxford, England), 28(12), 1647–1649. <https://doi.org/10.1093/bioinformatics/bts199>
11. ICTV (2024). The ICTV report on virus classification and taxon nomenclature. <https://ictv.global/report>.
12. Katoh, K., & Standley, D. M. (2013). MAFFT multiple sequence alignment software version 7: improvements in performance and usability. Molecular biology and evolution, 30(4), 772–780. <https://doi.org/10.1093/molbev/mst010>
13. Capella-Gutiérrez, S., Silla-Martínez, J. M., & Gabaldón, T. (2009). trimAl: a tool for automated alignment trimming in large-scale phylogenetic analyses. Bioinformatics (Oxford, England), 25(15), 1972–1973. <https://doi.org/10.1093/bioinformatics/btp348>
14. R: A Language and Environment for Statistical Computing (R Project, 2022).
15. Oksanen, J., Simpson, G., Blanchet, F.G., Kindt, R., Legendre, P., Minchin, P.R., O'Hara, R.B., Solymos, P., Stevens, M.H.H., Szoecs, E., Wagner, H., Barbour, M., Bedward, M., Bolker, B., Borcard, D., Carvalho, G., Chirico, M., De Caceres, M., Durand, S., Evangelista, H.B.A., FitzJohn, R., Friendly, M., Furneaux, B., Hannigan, G., Hill, M.O., Lahti, L., McGlinn, D., Ouellette, M.-H., Ribeiro Cunha, E., Smith, T., Stier, A., Ter Braak, C.J.F., Weedon, J. (2024). vegan: Community Ecology Package. R package version 2.6-7. Available from <https://github.com/vegandevs/vegan>
16. Csárdi G, Nepusz T, Traag V, Horvát S, Zanini F, Noom D, Müller K (2024). igraph: Network Analysis and Visualization in R. doi:10.5281/zenodo.7682609, R package version 2.0.3, <https://CRAN.R-project.org/package=igraph>.
17. Abatzoglou, J. T., Dobrowski, S. Z., Parks, S. A., & Hegewisch, K. C. (2018). TerraClimate, a high-resolution global dataset of monthly climate and climatic water balance from 1958-2015. Scientific data, 5, 170191. <https://doi.org/10.1038/sdata.2017.191>
18. Klein Goldewijk, K., Beusen, A., Doelman, J., and Stehfest, E.: Anthropogenic land use estimates for the Holocene – HYDE 3.2, Earth Syst. Sci. Data, 9, 927–953, <https://doi.org/10.5194/essd-9-927-2017>
19. IUCN. 2024. The IUCN Red List of Threatened Species. Version 2024-2. https://www.iucnredlist.org. Accessed on 25/07/2024.
20. Venables WN, Ripley BD (2002). Modern Applied Statistics with S, Fourth edition. Springer, New York. ISBN 0-387-95457-0, <https://www.stats.ox.ac.uk/pub/MASS4/>.
21. Bartoń, K. (2024). MuMIn: Multi-Model Inference. R package version 1.48.4. Available from <https://CRAN.R-project.org/package=MuMIn>
22. Bodenhofer, U., Bonatesta, E., Horejš-Kainrath, C., & Hochreiter, S. (2015). msa: an R package for multiple sequence alignment. Bioinformatics, 31(24), 3997-3999. DOI: 10.1093/bioinformatics/btv494


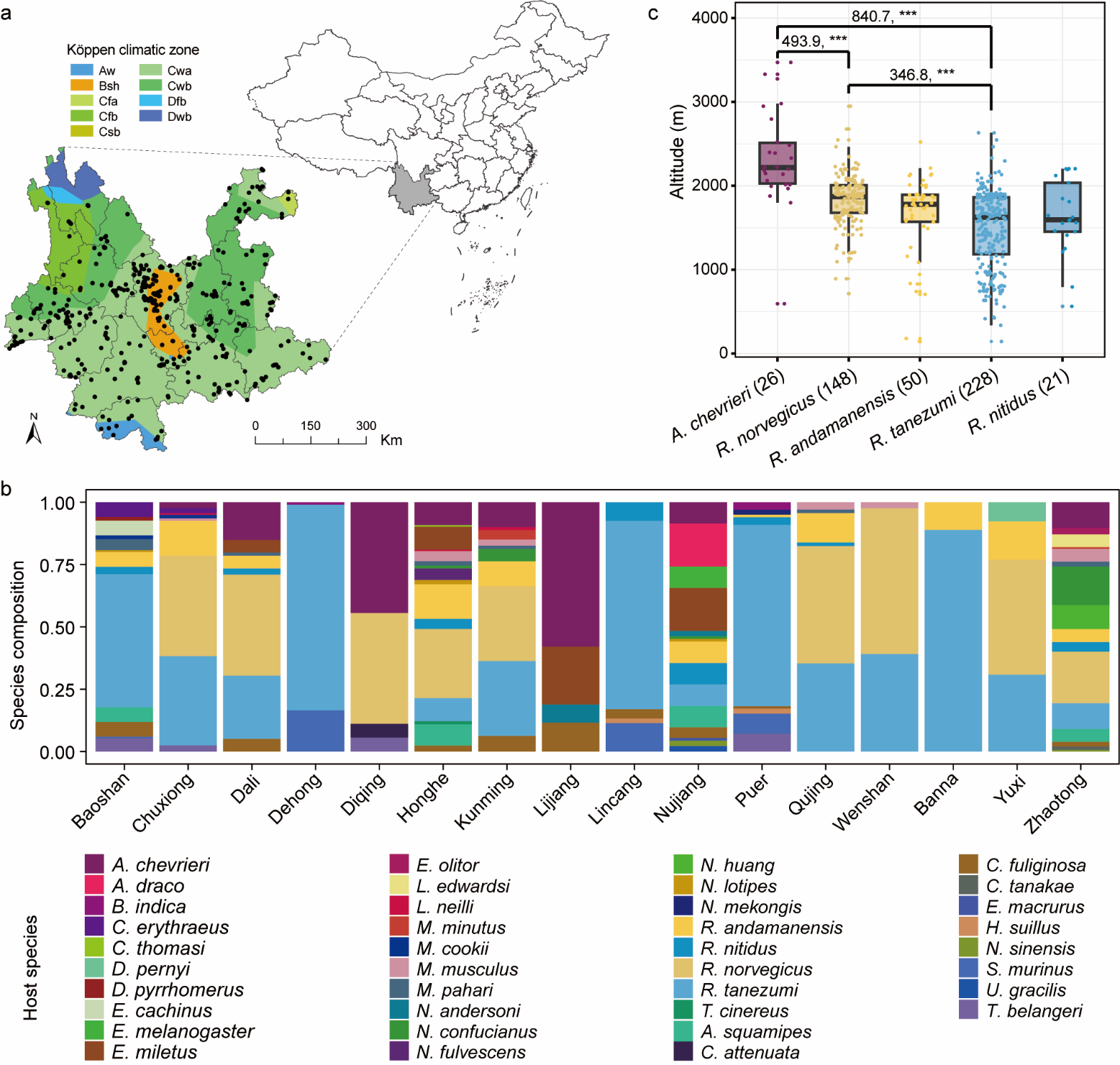


**Figure S1. Climatic zones and mammalian species composition in this study**. **(a)** Climatic zones within the sampling region, classified according to the Köppen climate classification system, with black points indicating sampling sites. The basemap shapefile was sourced from Tianditu (https://cloudcenter.tianditu.gov.cn) under review number GS (2024) 0650. **(b)** Composition of mammalian species in samples collected across various prefectures. **(c)** Boxplot depicting the altitude distribution (meters) of specific rodent species, including only those five distinct mammals collected from more than 20 different sites. Review drawing number: GS 京(2025)0083号.


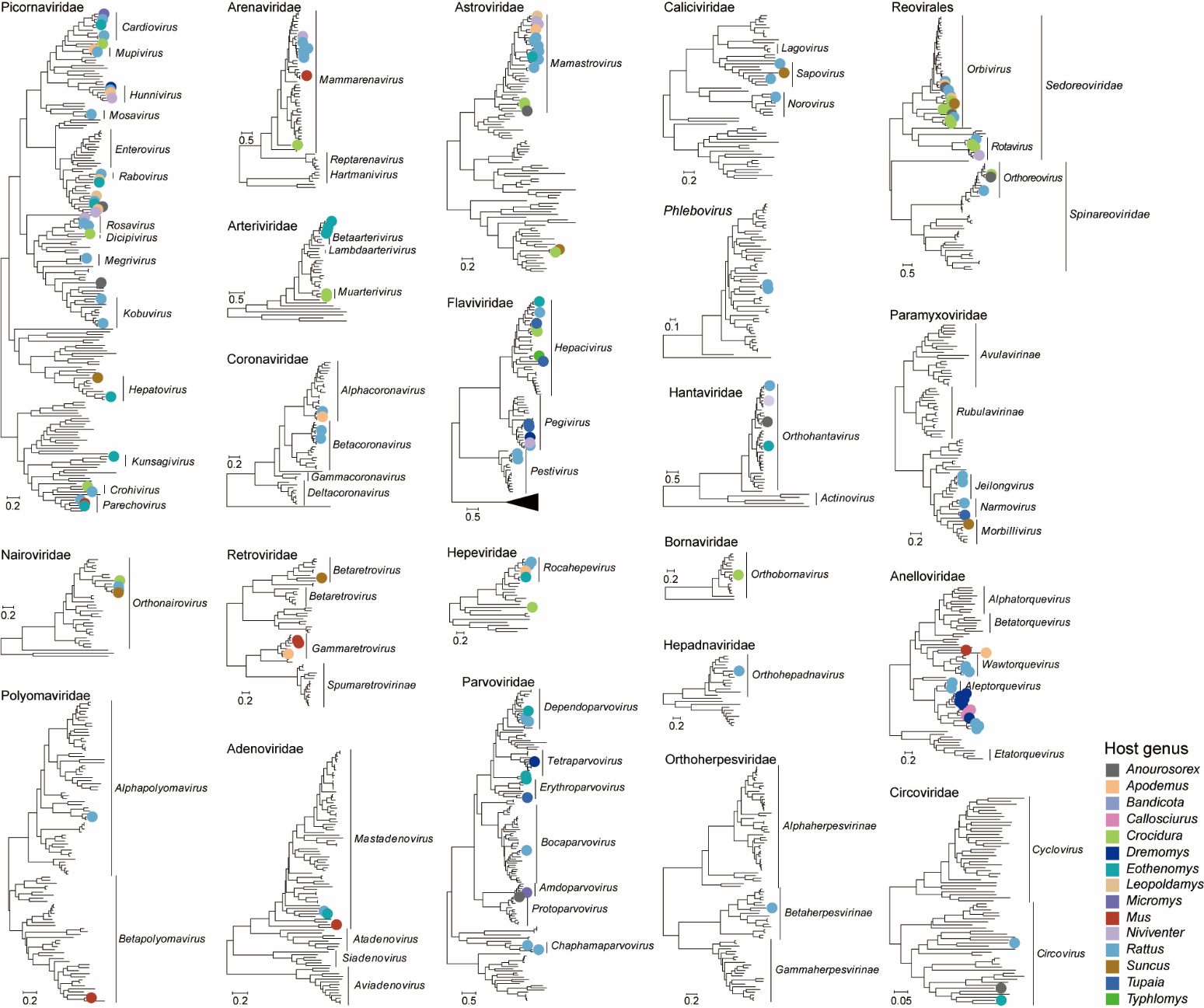


**Figure S2. Phylogenetic diversity of mammalian viruses from 23 families identified in this study.** Phylogenetic trees were estimated using a maximum likelihood method based on conserved protein sequences: RdRp for RNA viruses, reverse-transcriptase for the *Retroviridae*, the major capsid protein for the *Orthoherpesviridae*, LTAg for *Polyomaviridae*, ORF1 protein for the *Anelloviridae*, NS1 for the *Parvoviridae*, and DNA polymerase for other DNA viruses. Each tree is midpoint rooted with branch lengths scaled according to the number of substitutions per site. Viruses identified in this study are represented by dots, color-coded according to host genera, as detailed in the figure.


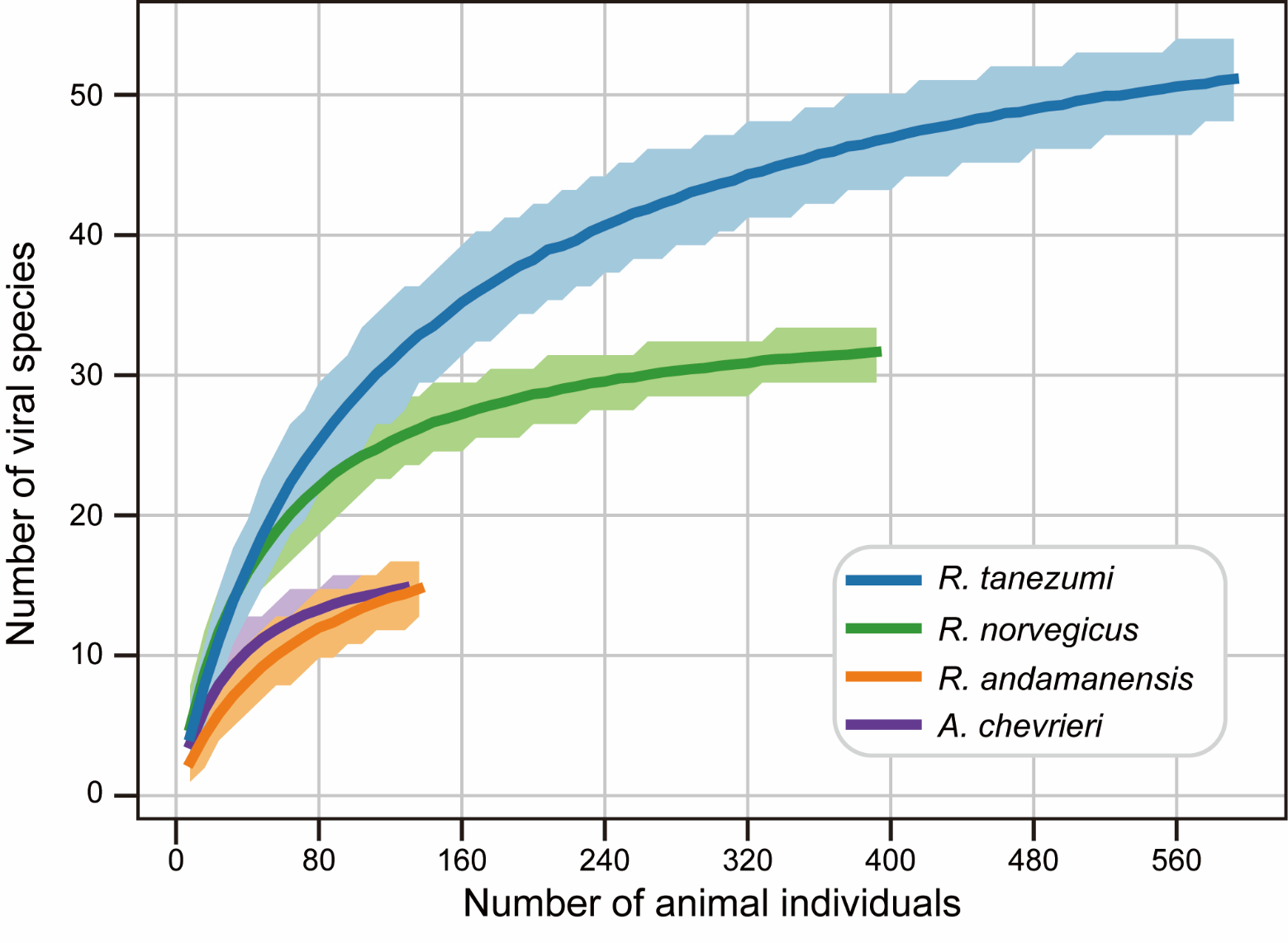


**Figure S3. Rarefaction curves for viral richness discovered in the well-sampled mammalian species.** A rarefaction analysis to evaluate the relationship between sample size and viral richness across mammalian species with more than 100 individuals (≥16 groups).


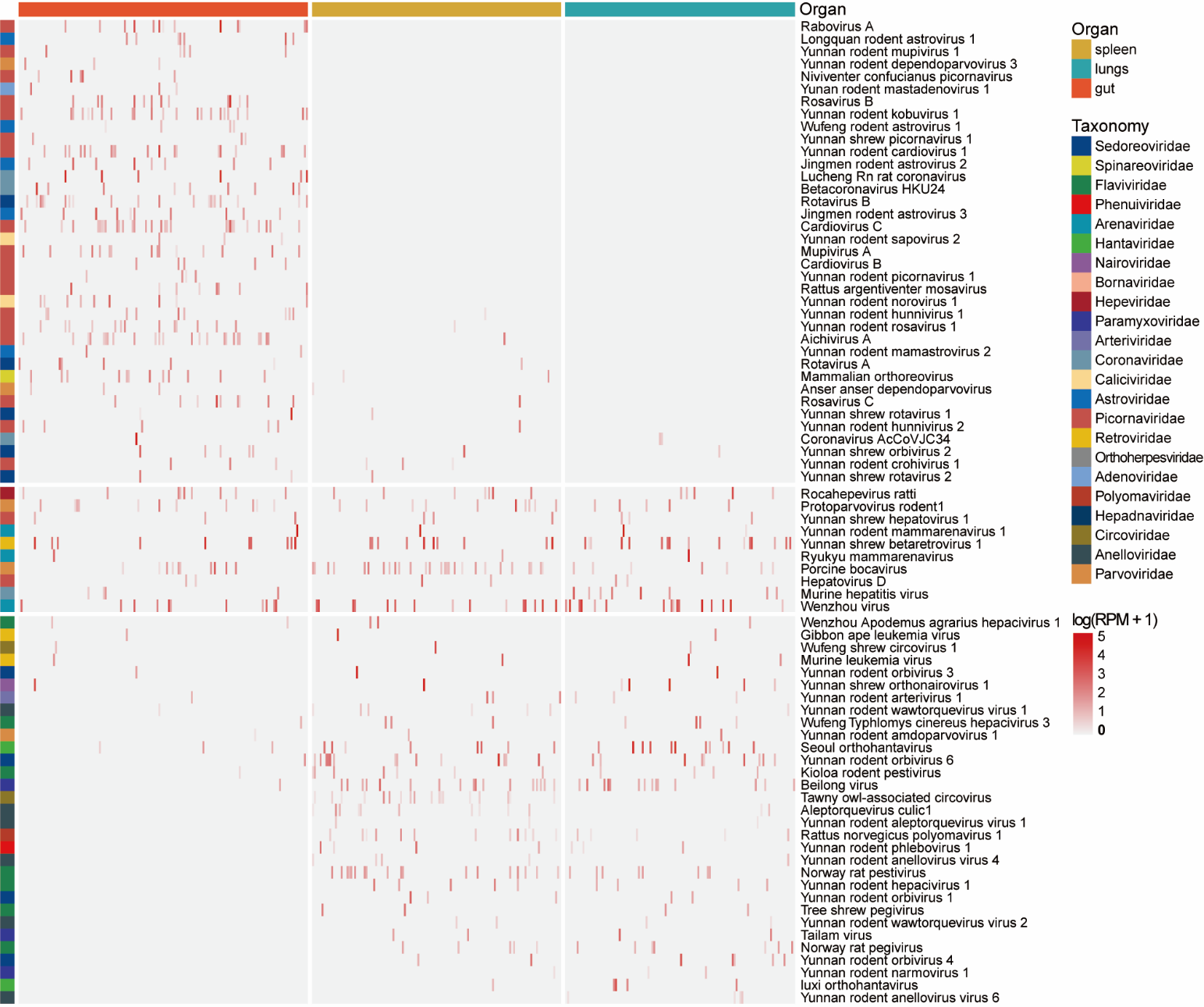


**Figure S4. Distribution of virus abundance across different organs.** Heatmap displays the abundance of each viral species across three organs (spleen, lungs, and gut) in mammalian hosts examined in this study. The arrangement of libraries and viruses is organized by Canonical Correspondence Analysis (CCA).


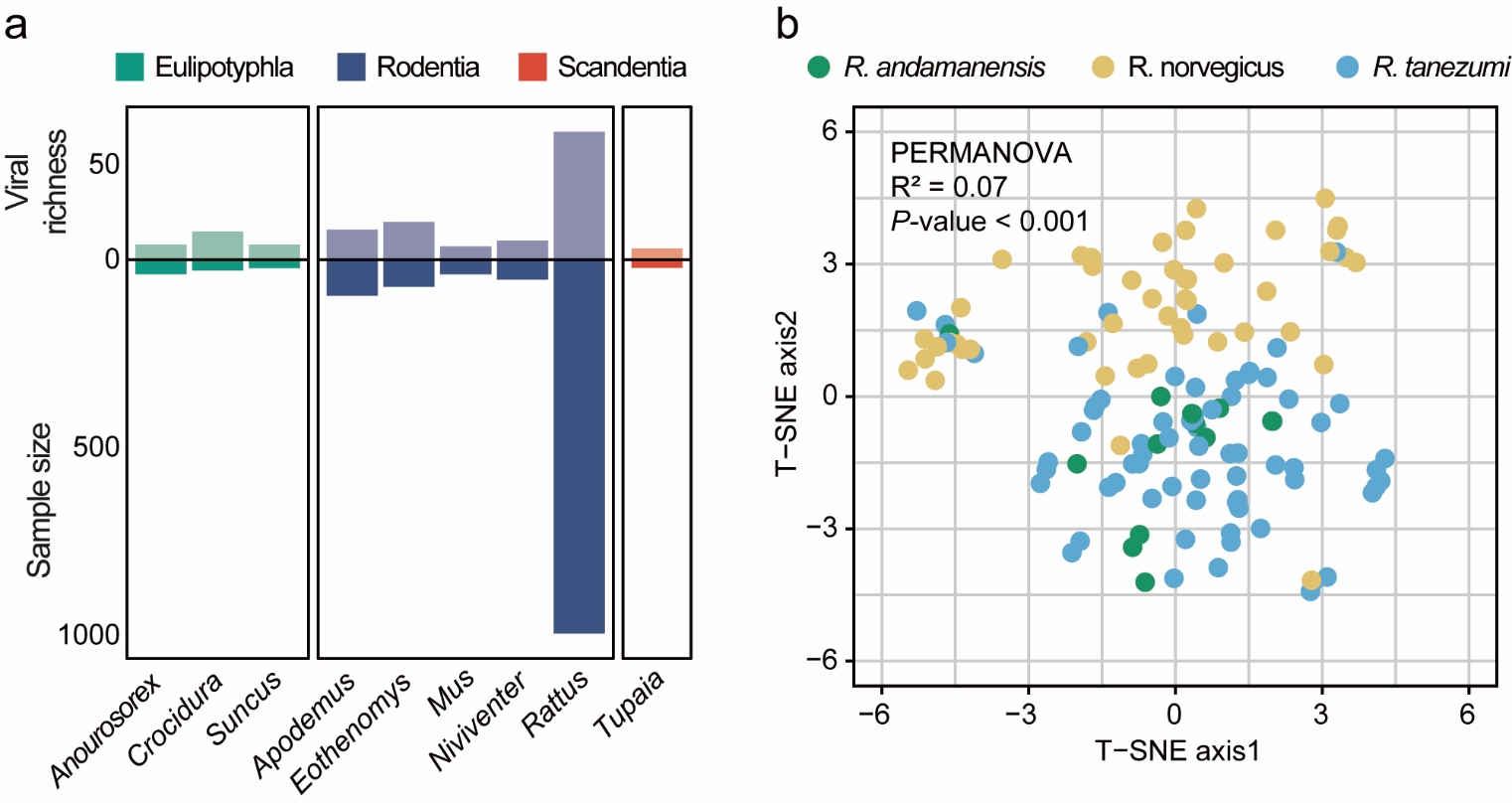


**Figure S5. Viral richness and composition across host taxa. (a)** Viral species identified in mammals of each genus of the orders Eulipotyphla, Rodentia, and Scandentia, including respective sample sizes. (**b)** t-SNE analysis showcasing the clustering of viral compositions among three rodent species within the genus *Rattus*: oriental house rats (*Rattus tanezumi*, n = 67), brown rats (*Rattus norvegicus*, n = 44), and Indochinese forest rats (*Rattus andamanensis*, n = 13). Statistical significance was assessed using PERMANOVA tests (two-sided) based on Jaccard distance with 1999 permutations.


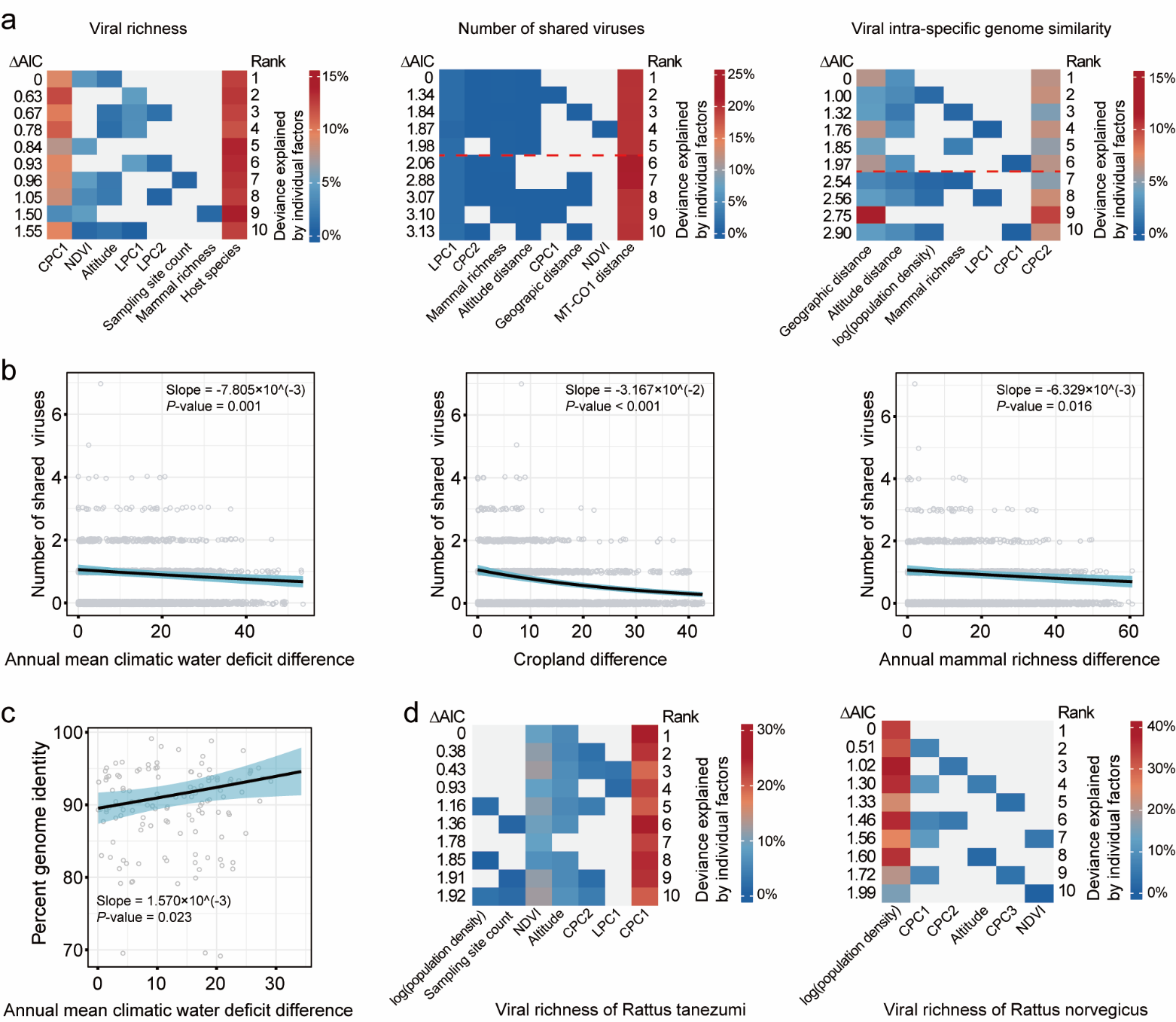
**Figure S6. Environmental and host factors affecting viral richness, composition, and intra-specific genomic diversity**. **(a)** Relative effects of mammal species, climate, and land-use characteristics on viral richness, composition, and intra-specific genomic diversity. These effects are quantified by explained deviance in generalized linear models. The top 10 models selected by AIC are displayed, with the red dashed line indicating models significantly supported (ΔAIC <2). **(b)** The impact of differences in annual mean climatic water deficit, cropland presence, and mammal richness on viral sharing between pairs of sample groups. **(c)** The impact of differences in annual mean climatic water deficit on intra-specific viral genome similarity. **(d)** Relative effects of environmental characteristics on viral richness within groups of oriental house rats (*R. tanezumi*) and brown rats (*R. norvegicus*).
